# What can evidence accumulation modelling tell us about human social cognition?

**DOI:** 10.1177/17470218231176950

**Published:** 2023-05-23

**Authors:** Samantha Parker, Richard Ramsey

**Affiliations:** School of Psychological Sciences, Macquarie University, Sydney, NSW, Australia

**Keywords:** Evidence accumulation model, computational model, social cognition, social attention

## Abstract

Evidence accumulation models are a series of computational models that provide an account for speeded decision-making. These models have been used extensively within the cognitive psychology literature to great success, allowing inferences to be drawn about the psychological processes that underlie cognition that are sometimes not available in a traditional analysis of accuracy or reaction time (RT). Despite this, there have been only a few applications of these models within the domain of social cognition. In this article, we explore several ways in which the study of human social information processing would benefit from application of evidence accumulation modelling. We begin first with a brief overview of the evidence accumulation modelling framework and their past success within the domain of cognitive psychology. We then highlight five ways in which social cognitive research would benefit from an evidence accumulation approach. This includes (1) greater specification of assumptions, (2) unambiguous comparisons across blocked task conditions, (3) quantifying and comparing the magnitude of effects in standardised measures, (4) a novel approach for studying individual differences, and (5) improved reproducibility and accessibility. These points are illustrated using examples from the domain of social attention. Finally, we outline several methodological and practical considerations, which should help researchers use evidence accumulation models productively. Ultimately, it will be seen that evidence accumulation modelling offers a well-developed, accessible, and commonly understood framework that can reveal inferences about cognition that may otherwise be out of reach in a traditional analysis of accuracy and RT. This approach, therefore, has the potential to substantially revise our understanding of social cognition.

## Introduction

Evidence accumulation models are some of the most successful frameworks used to account for human decision-making (Donkin & Brown, 2018). These models can account for choice and response time distributions across a range of decision-making tasks and have been used to advance our theoretical understanding of the way humans make decisions. These models are also commonly used as measurement tools, where accuracy and response times are translated into a set of latent variables that are assumed to underlie the decision-making process. These applications of accumulation models has shed light on the mechanisms that underlie a wide variety of cognitive processes including lexical decision-making (Wagenmakers et al., 2008), perceptual discrimination tasks (Ratcliff & McKoon, 2008), cognitive decline in ageing (Ratcliff et al., 2001, 2010), and attentional orienting (Parker et al., 2020a, 2020b, 2021). Yet despite their prevalence and success within cognitive psychology (for a review, see Ratcliff et al., 2016), there have been relatively few attempts to use these models to answer questions about human social cognition. The aim of this article is to explore ways in which the study of how humans attend, perceive, and interact within the social world could benefit from an evidence accumulation modelling approach.

We begin this article with a brief overview of the evidence accumulation modelling framework, including some of its past success within cognitive psychology. We then highlight five specific ways in which we believe the study of social cognition would benefit from an evidence accumulation modelling approach. We draw upon specific examples from the study of social attention, how humans orient in response to social information, to illustrate these points, as well as other domains of social cognitive research. In addition, we provide links to analysis code and data from our own work to provide a worked example of how an open-source evidence accumulation modelling architecture (Heathcote et al., 2019) can be adapted for use with experimental data from a study examining social cognition (Parker & Ramsey, 2023). Finally, we discuss some practical and methodological considerations when using an evidence accumulation model to study aspects of social cognition.

It is important to note that the goal of this article is not to provide a detailed or in-depth review of evidence accumulation modelling nor do we profess to have the relevant expertise to do so. Rather, our aim is to highlight how social cognition researchers may benefit from adopting this type of modelling approach within their own work. Therefore, our intended audience is anyone interested in social information processing who may not be familiar with cognitive modelling in general or evidence accumulation models specifically. Although our goal is to highlight how the evidence accumulation framework specifically can be used to shed new light on human social cognition, some of the discussed benefits also apply more widely to computational modelling in general. In other words, depending on the context, we expect social cognition research would benefit from the adoption of a wide range of computational approaches, which extend beyond evidence accumulation models. However, for brevity and clarity, we choose to focus on the modelling approach that we have experience using.

In making these suggestions, we draw upon our firsthand experience of using these models in our own experimental work, rather than an intricate knowledge of their development and formalisation (for more in-depth reading and review, see Donkin et al., 2009; Donkin, Brown, & Heathcote, 2011; Evans & Wagenmakers, 2020; Forstmann et al., 2016; Heathcote et al., 2019; Lewandowsky & Farrell, 2011). Ultimately, we suggest that an evidence accumulation modelling approach to studying social information processing has the potential to greatly enhance our understanding of the mechanisms that underpin human social cognition.

## Evidence accumulation modelling approach

### Overview

When attempting to understand human behaviour, it is common in psychology to draw inferences about psychological processes from performance on experimental tasks. One of the most common ways to measure performance is through the speed and accuracy with which we make a decision. For example, in a simple lexical decision task, an experimenter measures how fast and how accurately people are at detecting a word from a non-word when presented with a string of letters. Inferences are then drawn from differences in accuracy and reaction time (RT) across manipulations or participants.

Consider, however, a situation in which two participants show different patterns of speed and accuracy. One participant was very accurate but slow at detecting the letter string, while the other was less accurate but faster at responding. It is not possible in a separate analysis of accuracy and RT to determine which participant had superior ability on the lexical decision task or if they differed in ability at all. This is because accuracy and RT are incommensurable and therefore cannot be judged by a common standard. In other words, the separate analysis of speed and accuracy can limit the type of inferences drawn about the structure of cognition.

Although this example demonstrates the well-documented speed–accuracy trade-off phenomena, it is just one of the many ways in which accuracy and RT can interact. Evidence accumulation models have been developed to provide a better understanding of human decision making on speeded choice tasks (Brown and Heathcote, 2008; Ratcliff, 2002; Ratcliff and Rouder, 1998. They do this by providing a principled way of combining accuracy and RT distributions for correct and incorrect responses.

**Figure 1.**
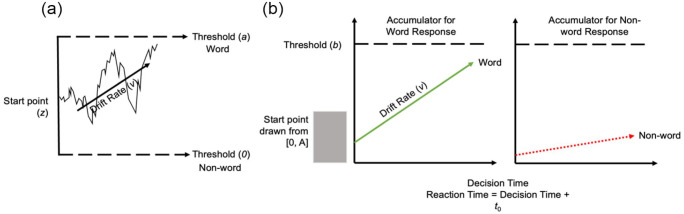
Schematic of (a) the diffusion decision model and (b) the linear ballistic accumulator. *Note.* This diagram represents the accumulation process for one trial of a lexical decision task for (a) the diffusion decision model (DDM), an example of a diffusion model and (b) the linear ballistic accumulator (LBA), an example of a race model. The participant is required to determine whether a letter string is a word or non-word. (a) The DDM is a single accumulator model where the relative evidence in favour of a response is sampled until sufficient evidence favours one response over another, the average speed of accumulation is known as the drift rate (*v*). The response made depends on which boundary (*a* for word response, *0* for non-word response) is reached first. (b) The LBA is a multiple accumulator model, meaning that there is a separate accumulator for each possible response. In this example, the correct response is “word” and incorrect response is “non-word.” The accumulator on the left is the accumulator for the correct “word” response, and the green line represents the rate at which evidence accumulates in favour of that response option, while the accumulator on the right is the accumulator for the incorrect “non-word” response and the dotted red line is the rate at which evidence accumulates in favour of this response option.

Evidence accumulation models refer to a set of computational models that all share a common framework to account for human decision-making. These models translate accuracy and RT into psychologically interpretable constructs that are assumed to underlie decision-making. Models under this framework broadly fall into one of two different categories. Diffusion models assume that when humans are making a decision between a number of alternatives, relative evidence accumulates in favour of one of these alternatives at a given rate over time, until enough evidence has accumulated in favour of one of these alternatives to trigger a decision (e.g., Ratcliff, 1978; Ratcliff & McKoon, 2008) (see Figure 1a for an example of the diffusion decision model (DDM); Ratcliff & McKoon, 2008). Race models, however, assume that evidence for each alternative accumulates independently, and the response is determined by the accumulator that crosses the threshold first (see Figure 1b for an example of a race evidence accumulation model, the linear ballistic accumulator (LBA) model; Brown & Heathcote, 2008).

Within these categories, there exist a variety of different evidence accumulation models (Brown & Heathcote, 2008; Ratcliff, 1978; Ratcliff & Rouder, 1998; Usher & McClelland, 2001); however, they all aim to provide estimates of four common aspects of the decision-making process. Drift rate is the rate at which evidence in favour of a decision accumulates. This measure is thought to reflect the signal-to-noise ratio provided by the stimulus. Drift rate is therefore able to quantify both the quality and quantity of evidence accumulating from a target. Faster drift rate is associated with more accurate and faster responding, while slower drift rate is associated with slower and more error prone responding. Threshold is the amount of evidence needed to trigger a response. This parameter measures response caution. Generally, higher thresholds are associated with higher response caution, that is, more accurate but slower responding. Lower thresholds, however, are associated with lower response caution, less accurate but faster responding. Start point noise is the amount of evidence in favour of a decision that exists at the outset of a decision. This parameter quantifies the a priori bias towards a response option. The more evidence in favour of a response at the outset of the decision, the higher the response bias in favour of that option before the stimulus is presented. Non-decision time is a measure of the time it takes to complete all processes that are thought to fall outside the decision-making process. This measure includes the time needed to encode a stimulus and make a motor response, such as pressing a button to indicate a response. Non-decision time is the time added to decision time to equal the response time.

### Model selection, fitting, and inferences

**Figure 2.**
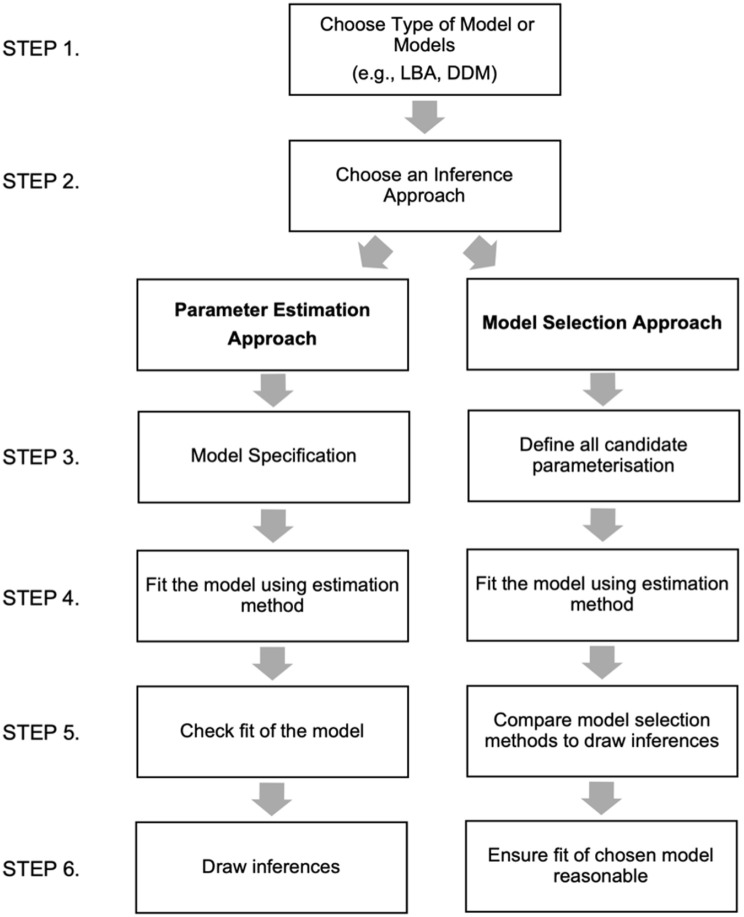
Summary of possible steps involved in an evidence accumulation modelling approach. *Note.* Step 1 involves choosing which type of evidence accumulation model (e.g., LBA and DDM) or several models (e.g., if performing a robustness check) that will be used in the analysis. It is important to ensure that the design of the experiment is suitable for the selected model. Step 2 involves selecting an inference approach; this can include parameter estimation, model selection, or both. Each inference approach involves different steps. The parameter estimation approach involves specifying how conditions will vary by parameters for the most complex model of interest (Step 3), fitting the model to the data using an estimation technique, such as maximum likelihood or Bayesian estimation (Step 4), assessing how well the model fits the data using statistical check or graphs (Step 5), and finally drawing inferences by examining how parameter estimates vary across conditions (including null-hypothesis testing or by assessing posterior distributions for estimates) (Step 6). The model selection approach involves first defining all possible parameterisations that will be investigated (Step 3), followed by fitting the models to the data using the same method outlined above (Step 4). Researchers then use model selection methods to compare models and draw inferences (Step 5). The final step is to ensure that the selected model from the comparison procedure provides a reasonable fit for the data (Step 6). Note that while this figure summarises some of the steps involved in the evidence accumulation modelling procedure, it is not exhaustive and other steps may include parameter recovery simulation studies or using a combination of these methods.

In general, an evidence accumulation modelling approach involves a number of steps. At a minimum, these steps include choosing an evidence accumulation model, selecting an approach by which to draw inferences from the modelling procedure, defining the parameterisation of the model/models, fitting the model using an estimation technique, assessing the fit of the model, and then drawing inferences (see Figure 2 for a summary of these steps). Within each step, researchers must make a number of decisions (see Dutilh et al., 2019).

Choosing a model involves first selecting the type of evidence accumulation model or several models to fit (e.g., LBA and DDM). It is important to ensure that the chosen model is appropriate for the design of the experiment. Following this, researchers should select an approach by which inferences will be drawn from the modelling procedure. Here, we outline two different inference approaches, parameter estimation and model comparison. Researchers may select one or both inference approaches.

Researchers using a parameter estimation approach to draw inferences should first specify the most complex model they are interested in. That is, how conditions will be allowed to vary, or not, by parameters. This specification can be informed by the prior literature, where constraints on the model are guided by previous studies. In the lexical decision task described above, for example, we might expect that the quality of the signal accumulating from a letter string to vary for words compared with non-words. We would therefore allow the drift rate parameter to vary by stimulus type (word vs. non-word).

The next step of the parameter estimation approach involves selecting the estimation method by which to fit the model to the data and estimate the parameters. There are a number of different methods of estimation that exist including both frequentist (Donkin, Brown, & Heathcote, 2011) or Bayesian approaches (Heathcote et al., 2019). Following this, how well the model predicts the observed data should then be assessed. Again, depending upon the estimation technique used to fit the model, there are a variety of ways to assess the degree to which the observed data and the predictions of the chosen model align. The most common approach is to plot the chosen models predictions alongside the observed data to ensure that the model is able to capture the major trends in the data (Voss et al., 2015). Inferences should only be made from the parameter estimates of the chosen model if that model is able to adequately mimic the observed data (Donkin & Brown, 2018).

There are a variety of approaches that can be used to draw inferences from parameter estimates about the underlying psychological mechanisms involved in a task (Dutilh et al., 2019). One traditional approach is to conduct null-hypothesis testing on resulting parameter estimates. This involves, for example, submitting estimates to a traditional analysis of variance (ANOVA) type analysis (Parker et al., 2020b). Alternatively, a Bayesian approach can be used to draw inferences from the posterior distribution of parameter estimates (Heathcote et al., 2019; Parker & Ramsey, 2023).

Researchers may also draw inferences using a model comparison or selection approach. Here, researchers aim to investigate which specification of a model provides the most parsimonious account of the data. This approach involves first defining candidate model parameterisations that will be compared. In the lexical decision task, for example, we may compare a model which allows thresholds to vary by stimulus type to one that allows drift rate to vary by stimulus type. After fitting the model using the estimation techniques outlined above, model selection methods can then be used to determine which model provides a better account of the observed data. Although an in-depth exploration of model selection methods is beyond the goals of this article, it is important to note that there are several different model selection techniques. These methods vary in several ways including ease of computation, similarity to, and from the Bayes factor (i.e., the relative likelihood of each model, given the data), as well as the ways in which they penalise model complexity. Ultimately, by comparing how different models predict observed data across conditions or participants, we can draw inferences about the mechanisms that underlie the processes. The final step in the model comparison approach involves ensuring that the selected best fitting models provide a reasonable account of the data (Donkin, Brown, Heathcote, & Wagenmakers, 2011).

While outside the scope of this article, it is worth mentioning that there are also several additional steps that researchers can take, both to explore different methodological approaches when they are unsure and to verify the results and outcomes of modelling procedures (Heathcote et al., 2015). Simulation, for example, involves running a model with a particular set of parameters to generate synthetic data. The general goal of simulating data is to clarify whether the model, together with the experimental data, can satisfy the researchers’ goal in identifying the cognitive mechanisms that underlie a psychological process. For example, researchers, who wish to determine the sample size necessary to detect an effect of interest, may simulate data from models that vary in sample size to inform their decisions. Simulation studies can also be used to verify the results of the modelling procedure in what is known as a parameter recovery study. Specifically, researchers can generate simulated data using the true model parameters, fit the model to the simulated data using the same method used to model the real data, and then determine whether the estimated parameters match the true data-generating parameters (Heathcote et al., 2019). This gives an estimate of the bias and uncertainty in the modelling method. A similar process, known as model recovery, can be used to determine the accuracy of discriminating between two models (see Heathcote et al., 2015).

### Past use and success

One particularly prevalent way of using evidence accumulation models within cognitive psychology is as a “measurement tool” for RT and choice data (Donkin et al., 2009; Dutilh et al., 2019; Evans & Wagenmakers, 2020). One of the strengths of this approach is that it allows research questions to be answered in terms of latent variables, rather than in the observed variables themselves (e.g., accuracy and RT) (Donkin et al., 2009; Evans, 2019; Evans & Wagenmakers, 2020). For instance, in the lexical decision example introduced earlier, application of an evidence accumulation model to the accuracy and RT data may demonstrate that Participant 1 had a higher threshold than Participant 2, but that there was no difference in drift rate between the two participants. This finding would lead to a substantially different understanding of the mechanisms that underlie lexical decision-making for these participants, than is available from a separate analysis of accuracy and RT. For example, one may conclude in a separate analysis of accuracy that Participant 1 was better at lexical decision-making than Participant 2, while an analysis of RT alone would lead to the opposite conclusion. In contrast, using a modelling approach, one can conclude that while the participants did not differ in their ability to complete the lexical decision task, but rather Participant 1 was more cautious when responding than Participant 2. Importantly modelling allows inferences to be drawn about the variables that underlie performance, as reflected in estimates of the four key parameters outlined above (Dutilh et al., 2019).

Evidence accumulation models are being used increasingly within psychology to investigate a wide range of cognitive processes. Perhaps one of the best examples of the modelling approach’s utility, however, comes from the ageing literature. One typical finding within ageing research is that response times on cognitive tasks, such as lexical decision-making, increase with age (Salthouse, 1996). This finding has led to the suggestion that there is an age-related decline in cognitive ability. Application of an evidence accumulation model to data from this task, however, revealed that rather than older adults performing more poorly than younger adults, differences in performance were actually due to differences in response caution (Ratcliff et al., 2001, 2010). That is, older adults were more cautious responders, requiring a higher level of evidence to trigger a decision, than younger adults. Importantly, this inference was not available from an analysis of accuracy or RT alone. That is, while a separate analysis of RTs led researchers to propose that there was a slowing down on cognitive tasks in older adults, accumulation modelling established that this effect was better characterised as more cautious responding.

This example is just one way in which the evidence accumulation approach has allowed researchers to draw inferences that were unavailable in traditional analyses (White & Kitchen, 2022). In our own work within the vision literature, we have used an evidence accumulation approach to investigate the relationship between covert and overt shifts in attention (Parker et al., 2020a, 2020b, 2021). Unlike studies that analyse accuracy and RT separately, in adopting this approach our results revealed covert attention to not be modulated by the preparation of an eye movement once differences in response caution were accounted for. Importantly, this finding allowed us to conclude that contrary to previous studies, covert and overt attention both independently contribute to task performance, and furthermore that each type of orienting was most likely mediated by a distinct underlying mechanism.

While evidence accumulation modelling has traditionally been applied to lower level two alternative forced choice decisions, with objectively correct and incorrect responses, more recently this framework has been used to explore complex, higher-order decisions (Busemeyer et al., 2019; Heathcote & Matzke, 2022). These types of models, for example, are increasingly being used to account for value or preference-based decisions, such as those involved in risky choice (Mohr et al., 2017) or consumer decisions (Krajbich et al., 2012). That is, decisions that are not objectively right or wrong, but rather based upon the subjective preferences or values of the participant (Busemeyer et al., 2019). These types of decisions typically involve more complex evaluations than simpler perceptual or categorisation tasks. By adapting evidence accumulation models, researchers have begun to shed new light on the psychological processes that underlie these more complex types of decisions and differentiate between competing theories of how context may shape value-based decisions.

The ability of evidence accumulation models to account for more complex decisions is particularly relevant for researchers interested in social information processing. Judgements and decisions in social contexts are frequently preference or value based and may involve the evaluation of multiple pieces of information that evidence accumulation models have been shown to accommodate. Indeed, there has been a small selection of studies that have begun to apply the accumulation framework to understand the cognitive mechanisms that underlie complex social phenomena such as discrimination (Axt & Johnson, 2021), implicit association (Klauer et al., 2007), and the decision to use a firearm (Johnson et al., 2017; Pleskac et al., 2018). Axt and Johnson (2021), for example, applied the DDM to investigate the psychological mechanisms that underlie discrimination based on attractiveness. The authors found that during a judgement task, there was both an initial bias towards physically attractive people (start point effect) and a continued influence of attractiveness over the decision (drift rate effect). The authors also used the DDM to explore which mechanisms were affected by interventions known to reduce bias.

This study is just one example of how evidence accumulation modelling may be used to shed new light on the cognitive mechanisms that underlie complex decision-making that is typical of human social information processing. In line with studies that have applied accumulation modelling in a social context, we believe that greater use and knowledge of this modelling framework to understand social phenomena will be of significant benefit to social cognitive research. We outline several of these advantages below.

## How can the study of social cognition benefit from evidence accumulation modelling?

### Specificity of assumptions

Researchers studying social cognition are broadly interested in understanding the psychological processes that underpin how we perceive, interpret, and represent information about ourselves and others. Unsurprisingly, research questions within this literature are therefore diverse, varied, and complex. As a result, there is a tendency within studies of social cognition, as well as psychology more broadly, for researchers to rely upon verbal theories (Yarkoni, 2020), that is, theories that are formulated and expressed in linguistic terms.

Part of the problem with relying upon a verbal theory is that (1) it is typical for parts of the system or relationship between psychological processes to be poorly defined or vague and (2) they require interpretation (Smaldino, 2017). Consider, for example, a typical claim made within the domain of social attention that humans preferentially attend to social stimuli. This type of proposition is typical within psychological research, yet the precise meaning of this statement is unclear and open to interpretation. This statement, for example, in its current formulation, does not define what constitutes “social stimuli,” what categories of stimuli are compared in order to draw the conclusion that social stimuli is “preferentially attended,” and how attention is being measured? Although this type of ambiguity in verbal theories is prevalent throughout psychology, it is particularly the case within the domain of social cognition due to the complexity of phenomenon that is typically studied.

Theories within social cognition must often consider how different elements of the self and others interact together to produce behaviour, yet verbal models typically do not articulate how these different levels of system relate to each other. One of the consequences of vague verbal models is that often many different types of experimental data can be consistent with the theory.

In contrast, computational models, such as an evidence accumulation model, require researchers to be explicit about the parts of the system under inquiry and the relationship between those systems (Charpentier & O’Doherty, 2018; Guest & Martin, 2021; Hackel & Amodio, 2018). They do this by substantiating the verbal theory into a collection of mathematical relationships. There is growing recognition of the need for formal models within psychology broadly and social cognition specifically. Indeed, recently there has been an increase in the number of researchers using computational approaches within the study of social information processing and social neuroscience (Charpentier & O’Doherty, 2018; Hackel & Amodio, 2018; Lockwood & Klein-Flugge, 2021). Generally, however, computational approaches remain underused within the domain of social cognition.

The evidence accumulation modelling framework is one of the several computational approaches that could be applied to study human social cognition. Although a comprehensive comparison of these different approaches is beyond the scope of this article, an advantage of the evidence accumulation modelling framework specifically is that it is well developed and has been used extensively within the domain of cognitive psychology (Evans & Wagenmakers, 2020). Consequently, it is well established how each of the parameters of an evidence accumulation model relate to their underlying psychological processes (Dutilh et al., 2019). The benefit of this is twofold. First, when specifying a theory in terms of an evidence accumulation model, the definition of each variable and their relationship is clear and explicit. Second, in interpreting the results of the modelling procedure, there is a shared understanding within the literature as to how to interpret parameter estimates (Farrell & Lewandowsky, 2010). For example, it has been widely established that thresholds represent a measure of response caution, with lower thresholds associated with faster and more error prone responding and higher thresholds with more accurate but slower responding. Therefore, there exists a common understanding of how to interpret two conditions or participants who differ in terms of threshold. Adoption of evidence accumulation modelling allows model predictions and assumptions to be clearly specified, and in addition, there is a well understood and shared language in how to interpret the resulting parameter estimates.

This is exemplified within the context of social cognitive research by a study by Pleskac and colleagues (2018) that investigated the role of race in the first-person shooter task. This task is designed to investigate the role of stereotypes in decisions to shoot, with participants viewing a series of neighbourhood images until a target individual appears holding an object (e.g., gun or wallet). Participants must select whether to “shoot” or “not shoot.” In applying the DDM model to data from this task, the authors contrasted three specific and clearly defined hypotheses about how race may affect the decision to shoot. The authors hypothesised that race may create an initial bias to shoot (start point hypothesis), affect the rate at which evidence accumulates (drift rate hypothesis), or the quantity of evidence needed to respond (boundary separation hypothesis). The authors found that rather than create an initial bias, race affected the rate of accumulation and for some individuals the level of evidence. Importantly, application of the DDM framework meant that the studies predictions were explicit, and the results were interpretable using a shared language.

### Comparing across blocked task conditions or between-subjects

In many experiments designed to investigate social cognition, research questions may require comparisons be made across blocked conditions or between separate subject groups. There are many studies examining social processes, for example, that necessitate participants only be exposed to one level of a condition. Laidlaw and colleagues (2011) designed a study to explore how individuals orient in response to a live versus pre-recorded individual. To effectively manipulate social context, participants were allocated into two separate groups and exposed to only the live or pre-recorded condition. Eye movements and detection performance were then compared across these two separate groups.

Other research questions within the domain of social cognition, for example, examine how social information processing varies between distinct groups of individuals, including those that differ in gender, cultural identity, or trait levels. These types of research questions require a similar between-subject comparison.

Although this cross-task or between-group research design may be necessary to answer specific social research questions, the type of conclusions that can be drawn from a separate analysis of accuracy and RT is limited. This is because there is no way to combine these variables, in a typical analysis, into a single measure to quantify subject ability or task difficulty (Wagenmakers et al., 2007). Therefore, when conditions are held constant across blocks or groups of participants, there is no way to determine whether differences in performance are due to differences in a participant’s ability, differences in difficulty across task manipulations, or differences in response caution. The speed–accuracy trade-off explored at the beginning of this article is just one example of how a separate analysis of speed and accuracy is unable separate out effects due to participant ability from effects due to response caution. The same holds true when comparisons are made across blocked task conditions.

One particularly prevalent paradigm for researchers interested in social orienting is the gaze-cueing task. In this variant of the classic Posner cueing paradigm, the gaze direction of a centrally presented face directs attention towards (valid) or away (invalid) from the location of an upcoming target (Driver et al., 1999; Frischen et al., 2007). The speed and accuracy with which a participant detects a target preceded by a valid gaze cue relative to an invalid gaze cue (cueing effect) is taken as a measure of attention.

One long-standing question within this literature concerns the extent to which orienting in response to social cues is similar to, or distinct from, orienting to non-social stimuli. In pursuit of this question, it is typical for researchers to compare performance when attention is directed with a social (gaze cue) compared with non-social cue (arrow cue), where cue type is presented across separate blocks (Morgan et al., 2014; Ristic et al., 2007). In a design such as this, however, it is unclear to what extent differences across blocks represent true differences in orienting to social and non-social stimuli or strategic differences that can occur across blocked task designs. Imagine, for example, that responses to gaze-cued targets are more accurate but slower than responses to arrow-cued targets. In a typical analysis of accuracy or response time, it is unclear how to combine these two variables into one measure which can unambiguously quantify orienting. Consequently, it is not possible to establish whether the influence of gaze cues on spatial attention is different from that associated with arrow cues or whether differences in accuracy and RTs are due to strategic differences that can occur across blocked task conditions.

Evidence accumulation modelling, however, provides a principled way to combine speed and accuracy. By parameterising the decision-making process, participant sensitivity and response caution can be separated (Stafford et al., 2020). This in turn allows effects to be unambiguously compared across blocked task condition or between-subject groups.

**Figure 3.**
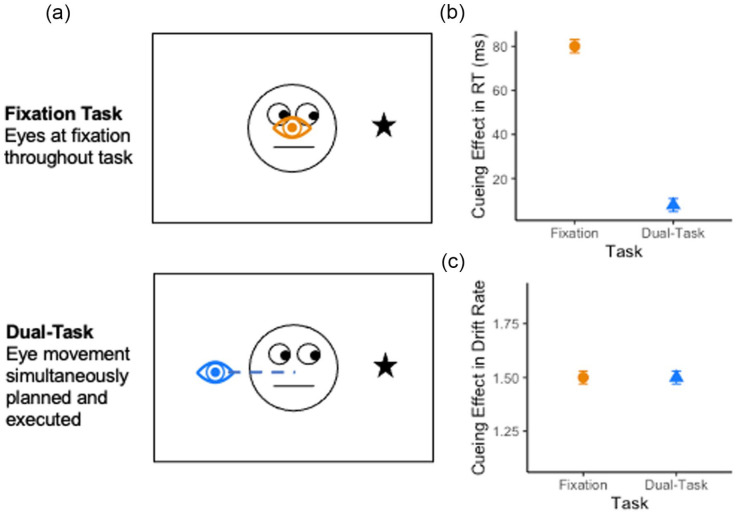
(a) Schematic of gaze cueing and saccadic dual-task and illustrative results in (b) reaction time and (c) drift rate. *Note.* (a) Schematic representing task design used in Parker and Ramsey (2023). The gaze-cueing effect was compared between a task in which one group of participants kept their eyes at fixation (fixation task) and a second task in which a separate group of participants simultaneously prepared eye movements (dual-task). (b) Illustrative results of typical findings for reaction time. Results show a modulation in the size of the cueing effect in reaction time measures. That is, there is a larger cueing effect on fixation (orange results) relative to dual-task trials (blue results). (c) Illustrative results in drift rate measures. Once differences in response caution are accounted for graph demonstrates that there is no difference in the magnitude of the cueing effect between fixation or dual-task trials. Drift rate is reported in standardised units. Note—the data displayed in (b) and (c) were created for illustrative purposes in this figure and do not reflect a summary of real experimental data.

We recently used this approach to investigate the relationship between spatial attention and the preparation of eye movements in social- and non-social-cueing tasks (Parker & Ramsey, 2023). One enduring question within the domain of attention research concerns the extent to which spatial attention is obligatorily coupled to the goal of an upcoming eye movement (Hoffman & Subramaniam, 1995; Klein, 1980; Posner, 1980; Rizzolatti et al., 1987). Evidence in favour of this proposition comes from studies that measure the cueing effect across two blocks of trials; one in which participants are required to maintain fixation while completing a perceptual discrimination task and another in which they are required to prepare and execute an eye movement (see Figure 3a). Any modulation in the magnitude of the RT-cueing effect across the two conditions is then typically interpreted as evidence to suggest that spatial attention is tied to the goal of an upcoming eye movement (see Figure 3b) (Born et al., 2013; Castet et al., 2006; Deubel, 2008; Hoffman & Subramaniam, 1995; Montagnini & Castet, 2007). Although task difficulty necessitates this type of blocked design, it is not possible using a typical analysis of accuracy and RT to separate out differences in response caution from task ability. This leaves open the possibility that differences in the cueing effect across the blocked conditions may reflect strategic differences across task, rather than the operation of spatial attention. For example, it is unclear whether a smaller cueing effect on dual-task trials compared with fixation trials is due to modulation in spatial attention during eye movement preparation, or higher response caution due to the difficulty of completing a dual relative to single-task trial.

To overcome this limitation, we combined an evidence accumulation model (Brown & Heathcote, 2008), with the gaze-cueing dual-task design (Parker & Ramsey, 2023). We found that, in contrast to studies that separately analysed accuracy and RT, once differences in response caution were accounted for, there was no difference in the magnitude of the cueing effect (see Figure 3c). That is, although the threshold parameters of the LBA model varied across task, the magnitude of the cueing effect in drift rate did not.

By extracting a measure of orienting that separates response caution from task difficulty, drift rate could be compared across blocked conditions and between groups. Using this approach, we were able to compare the contribution of covert and overt orienting as a function of perceptual task (high vs. low acuity) (Parker et al., 2021), discrimination target (oblique vs. vertical discrimination) (Parker et al., 2020a), and cue type (peripheral vs. central; Parker et al., 2020b) (social vs. non-social; Parker & Ramsey, 2023). Our results establish robust and consistent evidence to suggest that across both social- and non-social-cueing tasks, there is a separate and independent contribution of both spatial attention and oculomotor preparation to performance.

Importantly, in separating out effects of response caution from drift rate, evidence accumulation modelling offers a way to unambiguously compare across blocked task conditions or between-subjects to draw conclusions that would not otherwise be available in a typical analysis of accuracy and response times. As such, evidence accumulation modelling may be particularly valuable to the study of human social cognition, where blocked task and between-subject designs are prevalent.

### Quantifying the size of effects in latent variables

Another advantage of evidence accumulation modelling is that it allows the magnitude of underlying cognitive constructs involved in the decision-making process to be quantified. In extracting a measure of task ability that is independent of response caution, effects of interest can be unambiguously quantified and compared across manipulations, tasks, and subject groups.

Studies of social cognition are frequently concerned with the extent to which social factors modulate human decision-making behaviour. In the domain of gaze-mediated orienting, for example, a large number of studies have sought to assess how gaze-mediated orienting is modulated by social factors, such as gender, age, personality traits, social status, and emotional expression (for review, see Dalmaso et al., 2020). Typically, researchers then use null-hypothesis testing to compare performance on the gaze-cueing task between two groups or conditions that vary with respect to the social manipulation of interest. A significant difference in the accuracy or RT between these conditions is taken as evidence that attention is modulated by social factors.

Although useful, these types of analyses do not allow the precise extent to which orienting is modulated by these social factors to be quantified. Consequently, it is not uncommon for studies to report conflicting findings, with authors having little ability to compare between studies or reconcile results. There are several studies examining gaze-cueing, for example, that report unique orienting effects associated with social cues relative to non-social cues (Birmingham et al., 2012; Friesen et al., 2004; Hayward & Ristic, 2015; Marotta et al., 2012, 2018, 2019; Roman-Caballero et al., 2021). At the same time, however, there are an equal number of conflicting studies that report no or negligible differences between social and non-social cues (Brignani et al., 2009; Kuhn & Kingstone, 2009; Tipples, 2002, 2008). Inability to compare or quantify the degree to which orienting effects vary across paradigms and cueing manipulations has prevented the development of a consistent understanding of the mechanisms involved in social orienting to date.

Evidence accumulation modelling, in contrast, offers a way to reconcile these findings by quantifying the magnitude of an effect on the underlying cognitive construct. In adopting this approach in our own work, we recently quantified the cueing effect for social compared with non-social cues in a simple perceptual discrimination task where participants were required to maintain central fixation (fixation task Parker & Ramsey, 2023). Our results revealed that the quality of information accumulating from the target was five times larger when attention was directed with an arrow relative to a gaze cue. Interestingly, however, response caution was also 1.5 times higher in the arrow-cueing task compared with the gaze-cueing task. Results which suggest that not only did people extract a higher quality of signal when attention was directed with an arrow cue but that they also responded more cautiously, than when attention was directed with a gaze cue. Importantly, we were able to precisely measure the extent of these differences in a common, standardised, and unambiguous measure, drift rate.

Germar and colleagues (2014), similarly, applied the DDM to investigate how social influence affects perceptual judgements. In this study, participants were asked to determine whether a visual stimulus was more orange or blue. Prior to responding participants were shown the alleged responses of other participants. Half of the participants were told that these responses were relevant, while the other half were told they were irrelevant. In using the DDM, the authors were able to quantify the degree to which the majority response influenced perceptual judgements, reporting that while the majority response affected drift rates in both conditions, it had a significantly larger effect when participants were told the responses were task relevant.

The ability to quantify effects in this way provides us with an opportunity to compare studies that may be conflicting but also understand the psychological processes that underlie social cognition in a more nuanced and precise way. In the domain of social cognition, where researchers are particularly interested in how subtle manipulations in social factors may modulate information processing, this approach would allow a more in-depth understanding of human decision-making.

### Individual difference approach

Researchers are increasingly recognising that there is a discrepancy between the group-level results of social experiments conducted in the lab and how social phenomena occur in the real world (Hayward et al., 2017). Although gaze-cueing studies, for example, suggest that people automatically orient in response to the gaze direction of faces, others studies have suggested that in real-world situations people orient far less in response to gaze direction then expected by these studies (Gobel et al., 2015). One potential source of this discrepancy is that there is a large degree of individual variation in the way that humans attend to, represent and interact with social information. Averaging across individuals to examine group-level differences across conditions can fail to capture the diversity of how information processing occurs in the social world. Given this, there is growing interest within the social cognitive literature in understanding how individual differences and variability may underpin social processes (Heerey, 2015).

There are a variety of different methodological approaches to exploring individual differences. In the domain of social cognition, two designs are particularly common. First, studies compare performance across two groups that differ with respect to a variable of interest, including gender, age, or trait level (Bayliss & Tipper, 2005). Researchers interested in how social orienting changes as a function of age, for example, typically examine whether there are reliable differences in the gaze-cueing effect for a group of younger adults relative to a group of older adults (Slessor et al., 2010, 2008, 2016). The second approach involves researchers correlating performance on one task with trait levels on a second task. Researchers may, for example, correlate gaze-cueing effects with the degree to which participants display higher levels of autism traits on the autism-spectrum quotient measure. Both approaches, however, are limited. It is likely, for example, that under the first approach, in averaging across groups of individuals, variability in task performance is lost. While the latter approach requires a vast number of participants to have sufficient statistical power and is limited in the conclusion that can be drawn about causation between the variables.

Evidence accumulation modelling, however, offers a novel methodological approach to examining individual differences. Specifically, by comparing the best fitting model across individuals, we can assess how many and which participants display a specific effect and what aspect of the decision-making process characterises different patterns of performance between individuals (Evans & Wagenmakers, 2020). In the domain of social orienting, for example, there are some participants who do not display the classical gaze-cueing effect. Unlike a traditional analysis where these differences would be collapsed across a group of participants, a model selection approach allows us to understand what aspect of the decision-making process differs for these individuals. In a recent application of this approach to individual differences in the gaze- and arrow-cueing effects, a model comparison approach revealed that while the gaze-cueing effect was often characterised by a difference in non-decision time for most individuals, this was not the case for all individuals. There was a small subset of participants whose gaze-cueing effect was best characterised as a difference in start point (Alister et al., 2022). Importantly, these results highlight how the same pattern of accuracy and/or RTs in a task may not necessarily represent the operation of the same cognitive process for all individuals.

The model comparison approach, when used to examine individual differences, has the potential to reveal how the structure of cognition varies across participants. That is, by comparing which models provide the best fit for each individual, researchers can identify which cognitive components of a process are common to most participants and which are most likely to vary.

Similarly, in comparing how individuals differ with respect to their best-fitting model, we can quantify the degree of variability that exists within a task or psychological process. Imagine, for example, that there are a diverse range of different models that emerge for individuals completing a gaze-cueing experiment, while a smaller and more alike set of models emerge as providing the best fit for individuals completing an arrow-cueing task. We can conclude from this model comparison approach that there is a greater degree of individual variability in the psychological processes that underlie gaze-cueing relative to arrow-cueing. This novel approach to model fitting, therefore, has the potential to allow us to answer a new and diverse set of research questions that have, to date, been unavailable to researchers (Yarkoni & Westfall, 2017). Given the individual variability in responding often reported within studies of social information processing, a model comparison approach has the potential to significantly enhance our understanding of the psychological mechanisms that underlie social cognition including to what degree and in what ways these mechanisms vary across individuals.

### Reproducibility and availability of online tools

Reproducibility and replicability are core principles of scientific development. Over the past few years, however, the reproducibility of psychological research has come under increased scrutiny (Open Science Collaboration, 2015). The widely studied “replication crisis” in psychology has brought about many changes in the standard of psychological research and there has been greater emphasis on improving openness and transparency in psychological research and analysis (Munafo et al., 2017). Greater adoption of computational approaches, such as evidence accumulation modelling, can significantly improve reproducibility in social cognitive research for a number of reasons.

First, as outlined above, computational models allow researchers to formalise predictions and theories in terms of a set of mathematical equations. Formalisation makes it clear to other researchers, what is predicted and how the results should be interpreted (Farrell & Lewandowsky, 2010). Evidence accumulation models, specifically, are some of the most broadly applied frameworks within cognitive psychology; consequently, these particular models and their formalisation are widely understood by researchers (Evans & Wagenmakers, 2020). By virtue of making assumptions explicit and reducing individual interpretation, computational models are likely to improve reproducibility and replication of findings.

Second, computational models are generally formalised via computer code. This means that a researcher in another lab can easily implement the model via a common programming language and reproduce the predictions that arise from that model. This is particularly the case for evidence accumulation modelling, where several well-developed and freely available tools exist. These include EZ diffusion (Wagenmakers et al., 2007), fast-dm (Voss & Voss, 2007), fast-dm-30 (Voss et al., 2015), Diffusion Model Analysis Toolbox (DMAT; Vandekerckhove & Tuerlinckx, 2008), Hierarchical Drift Diffusion Model (HDDM; Wiecki et al., 2013), Dynamic Models of Choice (DMC; Heathcote et al., 2019), and ggdmc (Lin & Stickland, 2020).

These frameworks and accompanying tutorials (Donkin, Brown, & Heathcote, 2011; Heathcote et al., 2019; Voss et al., 2015) allow researchers without expertise in computational modelling to fit and implement complex evidence accumulation models to their own experimental designs. Indeed, within our own work, we have used the DMC tutorials and architecture (Heathcote et al., 2019) to fit the LBA to data collected from an experiment which investigated the relationship between covert and overt orienting during social and non-social cueing (Parker & Ramsey, 2023). Open access to evidence accumulation tools and resources, such as the DMC tutorials, is particularly valuable given the complexity and expertise required to run, fit, and understand the evidence accumulation modelling process. In addition, our own analysis code provides an example of how the DMC architecture can be adapted to fit an evidence accumulation model to real experimental data (see https://osf.io/fe9ds/ for data and code).

## Considerations when using evidence accumulation modelling to study social cognition

### Practical considerations: trial numbers and error rates

There are several practical considerations that researchers intending to use evidence accumulation modelling should consider in designing an experiment suitable for modelling. In the context of studies of social cognition, these considerations may carry implications for the type of research questions that can be pursued and through what experimental design. Two important considerations for researchers are the number of trials needed for accurate modelling and error response rates (Voss et al., 2015).

The number of trials necessary for accurate model fitting and parameter estimation is a central question for researchers using evidence accumulation models. It is typical for modelling studies, for example, to have a very large number of trials per condition (>1000 trials) (Ratcliff & Rouder, 1998). This is because simulation studies generally report that higher trial numbers lead to higher accuracy in parameter estimation (Ratcliff & Tuerlinckx, 2002; Vandekerckhove & Tuerlinckx, 2007). Other studies examining trial numbers from 20 to 150 per condition report improvements in parameter estimation as trial numbers increase (Ratcliff & Childers, 2015; Wiecki et al., 2013). Although high trial numbers for modelling studies appears to be accepted as the broad convention, Lerche and colleagues (2017) identified a number of key recommendations for trial numbers based upon distinct criteria, including which parameter estimates were being compared.

Similarly, in addition to having an adequate number of trials per participant, participants must also make enough errors (Heathcote et al., 2019; Voss et al., 2015). This is because it is generally thought to be difficult to obtain precise parameter estimates in designs with low error rates. Therefore, experimental designs that are to be fit with an evidence accumulation model are typically designed to elicit a reasonably high error rate, such as an error rate of 20% as a rough heuristic. More recent research, however, has found that for some models, such as the diffusion model, estimation remains valid even as accuracy approaches 100% (Evans, 2020). In addition, novel model variants have been shown to have robust measurement properties for some tasks with traditionally high accuracy, such as the Flanker task (Evans & Servant, 2022). It is therefore possible, even in situations in which error rates remain low, that evidence accumulation models can be used to draw inferences about underlying mechanisms. A related consideration is that evidence accumulation models have typically been designed to account for behaviour on speeded tasks (e.g., less than 1–2 s) (although see Lerche & Voss, 2019). It is likely that some experiments investigating more complex aspects of social cognition may not have response times that fit within these time frames, and therefore, accumulation modelling approaches may not be suitable.

Given these considerations, it is apparent that not all research questions will lend themselves to a design that facilitates evidence accumulation modelling. This may be particularly true for studies which aim to understand social phenomena. One reason for this is that some research questions within the social cognitive literature necessarily require participants be exposed to only one experimental condition, or to a stimulus once, to preserve the salience of the manipulation. Colombatto and colleagues (2020), for example, investigated how the magnitude of the gaze-cueing effect was modulated by the perceived intention of a gaze shift. To do so, participants in one group were exposed to an animation where the gaze shift appeared to occur as an unintentional deflection away from another person, while a separate group of participants viewed an animation where the gaze shift appeared to be made intentionally. Importantly, to ensure that the deflection manipulation was salient participants completed one trial of the experiment only. These types of social manipulations are not possible in a design which necessitates many trials per condition, as repetition of the manipulation may compromise its validity.

Similarly, other paradigms used to investigate social information processing may have low error rates. The gaze-cueing paradigm, for example, one of the most widely employed tasks used to investigate social attention, typically reports very high accuracy rates. As a result, studies tend to report response times only. It is typically thought that evidence accumulation modelling requires a sufficiently high error rate, somewhere in the order of 65%–95%, to allow for accurate parameter estimation (Voss et al., 2015). Consequently, to be compatible with this general guideline to evidence accumulation modelling, this type of task must be modulated to increase difficulty. In our own work, we achieved this by measuring performance on a perceptual discrimination task that requires high visual acuity for completion and included an adaptive staircase procedure to ensure perceptual discrimination performance was not at ceiling (Parker & Ramsey, 2023). Of course, it would be important to check that by adapting a paradigm in this way, unintentional confounds or other limitations are not baked into the experimental design. Alternatively, for designs that require low error rates, simulation studies may assist researchers in determining whether and which models are robust to violations in these general heuristics.

Another practical consideration for researchers interested in applying evidence accumulation models to real experimental data concerns computation time. Although modern computing and the availability of online tools have greatly increased the accessibility of these methodologies, fitting and estimating parameters can still take a significant amount of time and computing resources to complete. This is particularly true when compared with traditional analyses. For example, depending on the complexity of the model and the amount of data, model building can take many hours or days per model.

Importantly, although the discussion above explores some of the practical considerations that those wishing to pursue accumulation modelling should consider, it is not intended to be followed as a set of strict rules. Rather, our motivation is to highlight the considerations that we believe may be relevant to social cognition researchers, and outline some approaches, such as simulation studies, that could be used to address these issues.

### Sources of data

Evidence accumulation models have been developed to account for speeded choice responses. Specifically, these frameworks provide an explanation for the correct and incorrect response time distributions when participants make a response among a range of choices. Consequently, these models are limited to designs in which accuracy and response times are collected. However, accuracy and RT are just two measures of responding that can be gathered about decisions. Indeed, there is a vast array of additional information that can be collected and measured in studies of decision-making broadly, and the study of social information processing specifically, including neuroimaging data (although see Forstmann et al., 2016), survey and questionnaire data, decision confidence ratings, eye movements patterns, and pupil dilation. Currently, the literature is limited in ways to incorporate or account for these additional data in typical evidence accumulation models (Evans & Wagenmakers, 2020).

Consequently, evidence accumulation models are typically only applied to paradigms that collect similar sources of data, specifically accuracy and RT measures, and tend to have similar designs. Researchers must also be mindful that the assumptions that underlie these models and the theoretical inferences that follow from their use are therefore not constrained by additional sources of data (Evans & Wagenmakers, 2020). This may limit the types of conclusions that can be drawn from a study employing evidence accumulation modelling.

It should be noted, however, that there have been several recent innovations to evidence accumulation models that have enabled additional sources of data to be accounted for (Kvam et al., 2022; Turner et al., 2015; Vandekerckhove, 2014). The neural drift diffusion model (NDDM), for example, has been developed to simultaneously model neural and behavioural data at the single trial level and has shown that by incorporating neural data, it can provide a better prediction for behavioural data (Turner et al., 2015). Similarly, other innovations include the development of a cognitive model that can provide a unified account for both discrete and continuous responses (Kvam et al., 2022), as well as a model that can account for both behavioural and self-report data (Vandekerckhove, 2014). Given the diversity of experimental methods employed in studies of social cognition, as well as the types of measures collected, these innovations may be particularly relevant to social cognitive researchers.

### Generality of the model

There is no doubt that all computational models provide simplified accounts of psychological processes. Indeed, this simplification of a process is one of the most significant advantages to adopting a computational model. However, this simplicity also means that evidence accumulation models will always fail to capture and account for the entire process involved in social information processing (Navarro, 2021).

Formal computational models are necessarily simplified accounts of psychological processes. The benefit of this simplification is that these models are explicit in their assumptions, both in terms of what is defined by the model and by what is not defined by the model. Therefore, any conclusions from a model, while inherently flawed, also allow us to fine-tune theories by specifically determining in what ways the model differ from reality (Smaldino, 2017). Evidence accumulation models, while providing a good general description of decision-making, by design, do not account for the range of processes involved in social cognition. Further development and adaptation of these models may be necessary to provide more specific accounts of social cognition. Indeed, we believe this is further reason why the study of social information processing would benefit from greater use of these types of computational models. One avenue for future research, for example, is for experts in social cognition and experts in evidence accumulation modelling to work together to develop specific adaptations to models that are appropriate for social cognitive tasks.

## Conclusion

Evidence accumulation models have been used throughout cognitive psychology for the better part of 40 years. As a measurement tool, these models have been shown to provide insight into a range of phenomena that would otherwise be unavailable in a traditional analysis of accuracy and/or response times. In this article, we have reviewed five ways in which we believe the application of the evidence accumulation modelling framework would benefit the study of social information processing, drawing upon examples from the study of social attention to illustrate these points. Finally, we reviewed several considerations that researchers planning to implement this computational approach should consider, including ways in which this approach may be limited. Ultimately, from our perspective as researchers interested in social information processing, evidence accumulation modelling has the potential to enhance our understanding of human social cognition in new and important ways.
